# Spidroin striped micropattern promotes chondrogenic differentiation of human Wharton’s jelly mesenchymal stem cells

**DOI:** 10.1038/s41598-022-08982-8

**Published:** 2022-03-22

**Authors:** Anggraini Barlian, Dinda Hani’ah Arum Saputri, Adriel Hernando, Candrani Khoirinaya, Ekavianty Prajatelistia, Hutomo Tanoto

**Affiliations:** 1 School of Life Sciences and Technology, Bandung Institute of Technology, Bandung, West Java 40132 Indonesia; 2 Research Center for Nanosciences and Nanotechnology, Bandung Institute of Technology, Bandung, West Java 40132 Indonesia; 3 Faculty of Mechanical and Aerospace Engineering, Bandung Institute of Technology, Bandung, West Java 40132 Indonesia

**Keywords:** Mesenchymal stem cells, Stem-cell differentiation, Biomaterials - proteins

## Abstract

Cartilage tissue engineering, particularly micropattern, can influence the biophysical properties of mesenchymal stem cells (MSCs) leading to chondrogenesis. In this research, human Wharton’s jelly MSCs (hWJ-MSCs) were grown on a striped micropattern containing spider silk protein (spidroin) from *Argiope appensa*. This research aims to direct hWJ-MSCs chondrogenesis using micropattern made of spidroin bioink as opposed to fibronectin that often used as the gold standard. Cells were cultured on striped micropattern of 500 µm and 1000 µm width sizes without chondrogenic differentiation medium for 21 days. The immunocytochemistry result showed that spidroin contains RGD sequences and facilitates cell adhesion via integrin β1. Chondrogenesis was observed through the expression of glycosaminoglycan, type II collagen, and SOX9. The result on glycosaminoglycan content proved that 1000 µm was the optimal width to support chondrogenesis. Spidroin micropattern induced significantly higher expression of SOX9 mRNA on day-21 and SOX9 protein was located inside the nucleus starting from day-7. COL2A1 mRNA of spidroin micropattern groups was downregulated on day-21 and collagen type II protein was detected starting from day-14. These results showed that spidroin micropattern enhances chondrogenic markers while maintains long-term upregulation of SOX9, and therefore has the potential as a new method for cartilage tissue engineering.

## Introduction

Articular cartilage has a limited intrinsic capacity to regenerate spontaneously after injury, often leading to pain and disability. The nature of articular cartilage that does not have blood vessels, neurons, and lymphatic system hinders the repair and regeneration potential once injured^1^. Osteoarthritis (OA) is the pathology of articular joints most commonly associated with defects in cartilage such as osteochondral defects^2^. To address this issue, cellular therapy has been extensively invested in exploring a new paradigm for the treatment of many degenerative diseases, including OA^3^.

Mesenchymal stem cells (MSCs) have been proposed as a regenerative cellular therapy for degenerative musculoskeletal conditions like OA^1,2^. MSCs can differentiate into osteocytes, adipocytes, chondrocytes, and myocytes, making MSCs promising to use in regenerative approaches^4^. In this study, the MSCs source was obtained from the umbilical cord, specifically from human Wharton’s jelly connective tissue, hence the name human Wharton’s jelly MSCs (hWJ-MSC). Wharton’s jelly-derived MSCs are advantageous in several aspects including easily harvested from discarded umbilical cord obtained at birth, exhibit a high proliferation rate, can be expanded for many population doublings, hypo-immunogenic, and non-tumorigenic^5,6^. Therefore, it does not cause problems in terms of ethical consideration compared to the other sources of MSCs. Immune-modulation, one of the characteristics of hWJ-MSCs, can be an essential feature for promoting the use of these cells in cell-based therapy^7^.

Chondrocytes are usually obtained from differentiating MSCs in culture using chemical inducers, such as by using transforming growth factor-beta (TGF-β) superfamily, isoform bone morphogenetic protein (BMP), activin, and osteogenic protein-1^8^. However, this method has a high cost of production because it takes various growth factors to differentiate MSCs culture^9^. The alternative method to overcome this problem is by using mechanoinduction or mechanical stress^9^. Mechanical stress is needed in chondrogenesis to obtain cell-specific contact conditions or cell aggregation that leads to chondrocyte maturation^10^. In this research, a striped micropattern was used to trigger mechanical stress. Cell micropatterning comprises the use of a substrate with microscopic features that impose a defined cell adhesion pattern. It is a highly efficient method to investigate the sensitivity and response of a cell to specific microenvironmental cues^11^.

One of the micropatterning methods that have been widely used is microcontact printing (µCP). Microcontact printing is a particular type of soft lithography and replica molding procedure. In this process, an ink solution is transferred from an elastomeric mold or stamp to a substrate surface^12^. Several polymers have been used to perform microcontact printing procedure including poly(dimethylsiloxane) (PDMS), polyvinylpyrrolidone (PVP), poly(butylene terephthalate)-poly(tetramethylene glycol) (PBT-PTMG), agarose, polyetherimide (PEI), and poly(ether sulfone) (PES)^13^. Some hydrophobic polymers such as PDMS are not suitable for stamping polar molecules, especially for transferring biological ink (bioink) such as extracellular matrix protein^14^. The use of hydrophobic polymer stamps can result in the ink not sufficiently absorbed, and as a consequence, the ink cannot be transferred to the substrate. Therefore, in this study agarose which is a hydrophilic polymer was used. Agarose stamp is remarkably durable and can form a wide range of patterns using bioink on various substrates, for example, glass and plastic^15^.

Fibronectin has RGD motifs (tripeptide Arg-Gly-Asp) for integrin binding and allows cells to attach to the substrate, guides cell migration, and helps cells proliferate on the substrate^16,17^. Fibronectin is one of the components of the extracellular matrix and is considered the gold standard for micropattern ink using microcontact printing protocol^18^. Interestingly, spider silk protein (spidroin) is a natural material known to have RGD sequences^19^. RGD sequences that are present in *Argiope appensa* spidroin facilitate the attachment, spreading, and proliferation of hWJ-MSCs^20,21^. Previous studies have indicated that spidroin from *Argiope appensa* played an important role in the chondrogenic differentiation of MSCs^21,22^. Spidroin from *Argiope appensa* had an impact on scaffold compressive strength and induced chondrogenic differentiation of hWJ-MSCs^21^. Also, spidroin bioink was able to increase the glycosaminoglycan levels of hWJ-MSCs grown on micropattern substrate^22^.

However, the role of spidroin micropattern in chondrogenic differentiation has not been studied further. In addition, the exact pathway and mechanism in which micropattern exerts its effect remains unclear. The extend to which spidroin bioink can be used to substitute fibronectin for microcontact printing needs to be elaborate more, especially the effect on chondrogenic marker expression levels and the presence of RGD sequences in micropattern. This study aims to determine the role of spidroin striped micropattern in directing the chondrogenic differentiation of hWJ-MSCs.

## Material and methods

### Primary culture of hWJ-MSCs

Human Wharton’s jelly (hWJ) which was derived from the umbilical cord, was obtained from Bandung Maternity Hospital (RSKIA) in Bandung with informed consent from the donors and ethical clearance. Methods used in this study were carried out according to the relevant regulations and guidelines. All experimental protocols were approved by the Medical and Health Research Ethics Committee (MHREC) and recognized by Forum for Ethical Review Committees in Asia and the Western Pacific (FERCAP). The primary culture method used was the explant method. The samples were washed with 10% iodine solution, subsequently, the veins and arteries were then removed. hWJ was cut into 1 × 1 cm in size, then placed in a 100 mm tissue culture dish, and incubated at room temperature until these tissues were attached. The growth medium used in the primary culture contains Dulbecco's modified eagle medium (DMEM, Gibco), 10% fetal bovine serum (FBS, Gibco), and 1% antibiotic–antimycotic (Gibco). The primary culture's dish was then incubated at 37 °C 5% CO_2_ with medium replacement every 2–3 days^23^. Cells were passaged using Trypsin–EDTA 0.25% (Gibco) once they reached 80% confluence.

### Characterization of hWJ-MSCs

The hWJ-MSCs phenotype was determined using flow cytometry according to Human MSC Analysis Kit (BD Stemflow) protocol. A total of 5 × 10^5^ cells were collected for each sample. Afterward, 100 µL cells suspension were added into 20 µL mixing a solution of human mesenchymal stem cells (hMSC) positive cocktail (CD73-APC, CD90-FITC, and CD105-PerCP markers) and 20 µL PE hMSC negative cocktail (CD45, CD11b, CD19, and HLA-DR PE markers) then incubated in the dark condition for 30 min. Cells were washed with staining buffer, resuspended with 300–500 µL FBS, and analyzed on the flow cytometer. The hMSC positive cocktail was used to identify hMSC markers while the hMSC negative cocktail was used to detect potential contaminants.

hWJ-MSC multi differentiation assay was carried out to determine the potential of these cells to differentiate into chondrocytes, adipocytes, and osteocytes. A total of 1 × 10^4^ passage 5th cells were cultured on 24 well-plate with chondrogenic, adipogenic, and osteogenic medium (Stempro differentiation medium, Gibco). Then the cells were incubated at 37 °C 5% CO_2_ with medium replacement every 2–3 days. After 21 days, cells were fixed with 4% formaldehyde. Cells differentiation was observed by cell staining using Alcian blue for chondrocytes, Alizarin Red for osteocytes, and Oil Red O for adipocytes. The staining results were observed using phase-contrast microscopy.

### Spidroin bioink production

The solubilization of the spider silk to obtain spidroin bioink was based on the Hernando et al. (2021) method^22^. First, 0.08 gr of silk was added to 2.45 ml 95% ethanol, 3.84 ml dH_2_O, 3.92 gr CaCl_2_, and 3.84 gr urea. Then the solution was incubated at a high temperature for 20–24 h with constant stirring using a magnetic stirrer. After the silk had dissolved, the solution was transferred into a dialysis membrane. Dialysis was done against 5 mM Tris–Cl pH 8 overnight at 4 °C. Subsequently, the solution was centrifuged at 5,000 rpm for 10 min. The supernatant containing soluble proteins was collected. Final protein concentration was measured using NanoDrop spectrophotometer at 280 nm. The spidroin concentration after solubilization was typically varied between 1.1 and 1.5 mg/ml. This study used a spidroin concentration of 50 µg/ml, the same concentration as fibronectin used in the microcontact printing protocol. Finally, the soluble spidroin was sterilized using a 0.22 µm syringe filter.

### Spidroin characterization

The average viscosity and zeta potential of spidroin solution were then measured using Horiba Scientific Nanoparticle Analyzer SZ on the basis of dynamic light scattering technique. About 1 ml of spidroin solution was inserted into the cuvette using a syringe. All measurements were performed in triplicates at 25 °C. Results were reported as mean ± standard deviation (SD) (n = 3).

The molecular weight of spidroin was determined using SDS-PAGE. Soluble spidroin solution was mixed 1:1 with 2X Laemmli buffer and the mixture was heated to to 99 °C for 5 min. The final concentration of the spider silk protein and fibronectin was 40 µg/mL. After equilibrating the sample at room temperature, the mixture was pipetted into a well of a 5% stacking gel cast on top of a 8% resolving gel. ExactPro 5–245 kDa (Prestained Protein Ladder, 1st BASE) was used as protein standard as a reference to evaluate the size of the protein targets. Gel electrophoresis was performed using a chamber (Bio-Rad) and the module was filled with a 1X SDS-Running buffer. Electrophoresis was carried out applying 100 V for 20 min followed by 120 V for 40 min. After electrophoresis, the gel was stained with Coomassie blue solution (0.05% Coomassie brilliant blue R250, 50% (v/v) methanol and 10% (v/v) glacial acetic acid) for 20 min at room temperature and subsequently destained in a destaining solution (30% (v/v) methanol and 10% (v/v) acetic acid) overnight. The gel image was captured and analysed using Gel Doc EZ Gel Documentation System (Bio-Rad).

### Micropattern fabrication using microcontact printing (µCP)

**Figure 1:**
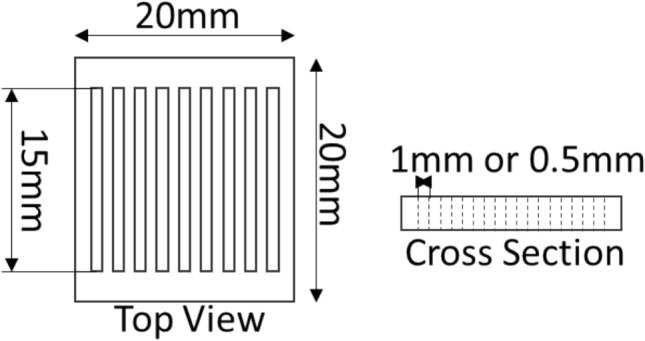
Mechanical drawing of acrylic master mold for agarose stamp production.

The micropatterning method was carried out using a microcontact printing (µCP) protocol with an agarose stamp to transfer the ink into the coverslip. The micropattern master mold was made from an acrylic board, laser cut to form two 20 × 20 mm squares equipped with 18 holes of 0.5 × 15 mm or 9 holes of 1 × 15 mm for 500 µm and 1000 µm, respectively (Fig. 1). Agarose 3% (w/v) was used to make the stamp using a master mold as a template, the resulting agarose stamp was then used for microcontact printing. The stamping process was in a sterile condition inside a biosafety cabinet. Before starting the stamping procedure, agarose and coverglass are sterilized by soaking with 70% alcohol under UV for 15 min. Subsequently, the agarose stamp was washed using phosphate buffer saline (PBS) 3 times and dried. 10 µl of fibronectin ink or spidroin ink (50 μg/ml concentration) was dripped in each micropattern agarose terrace. After the stamp absorbed the ink solution (usually within less than 5 min), the bioink on agarose was stamped onto the coverslip. The incubation period of 5 min was done for the fibronectin or spidroin to be completely transferred into the coverglass. Dried bioink on the coverslip will form striped patterns and hWJ-MSCs were ready to be seeded on micropattern^24^. A total of 5 × 10^4^ cells were seeded onto micropatterned coverglass (22 × 22 mm) with micropattern sizes of 500 µm, 1000 µm, and control (without micropattern stamping) then cultured for 21 days.

### Cell morphology using scanning electron microscopy

hWJ-MSCs that had been cultured for 3 days on a micropattern were washed with sterile PBS and then fixed using 2.5% glutaraldehyde in 0.1 M Na-Cacodylate, incubated at 4 °C for 24 h. Afterward, the sample was dehydrated with alcohol series (30%—100%) and dried using HMDS (hexamethyldisilazane). The samples were coated with gold and then observed by Scanning Electron Microscopy (Hitachi SU3500).

### RGD sequence immunocytochemistry

The micropatterned-substrates that have been stamped with spidroin bioink and cells that have been grown on micropattern for 7 days, were fixed with methanol-DMEM series (50%, 70%, 80%, 90%, 100%) at −20 °C followed by washing three times in PBS at room temperature. After fixation, cells were permeabilized in PBS-T (0.05% Tween 20 in PBS) for 20 min at room temperature. Cells were blocked from unspecific binding antibody with 3% Bovine Serum Albumin (BSA, in PBST) for 60 min. The primary antibody against the RGD sequence (ab224465, Abcam) was then added to the samples and incubated 4 °C overnight. The samples were washed with PBS three times and incubated in the secondary antibody (1:200 goat anti-rabbit IgG HNL Alexa Fluor 488 ab150077, Abcam) in a dark chamber for 120 min and counterstaining with 4′,6-diamidino-2-phenylindole (DAPI, Thermo Fisher) for 10 min followed by washing three times with PBS. The stained cells were observed with a confocal microscope (Olympus Fv 1200 confocal laser scanning microscope).

### Integrin β1 immunocytochemistry

Cells that have been grown on micropattern for 2 days, were fixed with methanol-DMEM series (50%, 70%, 80%, 90%, 100%) at −20 °C followed by washing three times in PBS at room temperature. After fixation, cells were permeabilized in PBS-T (0.05% Tween 20 in PBS) for 20 min at room temperature. Cells were blocked from unspecific binding antibody with 3% Bovine Serum Albumin (BSA, in PBST) for 60 min. The primary antibody against Integrin β1 (PA5-29,606, ThermoFisher Scientific) was then added to the samples and incubated 4 °C overnight. The samples were washed with PBS three times and incubated in the secondary antibody (1:200 Alexa Fluor 647, ab150115, Abcam) in a dark chamber for 120 min and counterstaining with 4′,6-diamidino-2-phenylindole (DAPI, Thermo Fisher) for 10 min followed by washing three times with PBS. The stained cells were observed with a confocal microscope (Olympus Fv 1200 confocal laser scanning microscope).

### Collagen type II immunocytochemistry

Cells that have been grown on micropattern for 7, 14, and 21 days were fixed with methanol-DMEM series (50%, 70%, 80%, 90%, 100%) at −20 °C followed by washing three times in PBS at room temperature. After fixation, cells were permeabilized in PBS-T (0.05% Tween 20 in PBS) for 20 min at room temperature. Cells were blocked from unspecific binding antibody with 3% Bovine Serum Albumin (BSA, in PBST) for 60 min. The primary antibody against Collagen type II (ab34712, Abcam) was then added to the samples and incubated 4 °C overnight. The samples were washed with PBS three times and incubated in the secondary antibody (1:200 goat anti-rabbit IgG HNL Alexa Fluor 488 ab150077, Abcam) in a dark chamber for 120 min and counterstaining with 4′,6-diamidino-2-phenylindole (DAPI, Thermo Fisher) for 10 min followed by washing three times with PBS. The stained cells were observed with a confocal microscope (Olympus Fv 1200 confocal laser scanning microscope).

### SOX9 immunocytochemistry

Cells that have been grown on micropattern for 7, 14, and 21 days were fixed with methanol-DMEM series (50%, 70%, 80%, 90%, 100%) at −20 °C followed by washing three times in PBS at room temperature. After fixation, cells were permeabilized in PBS-T (0.05% Tween 20 in PBS) for 20 min at room temperature. Cells were blocked from unspecific binding antibody with 3% Bovine Serum Albumin (BSA, in PBST) for 60 min. The primary antibody against SOX9 (ab3697, Abcam) was then added to the samples and incubated 4 °C overnight. The samples were washed with PBS three times and incubated in the secondary antibody (1:200 goat anti-rabbit IgG HNL Alexa Fluor 488 ab150077, Abcam) in a dark chamber for 120 min and counterstaining with 4′,6-diamidino-2-phenylindole (DAPI, Thermo Fisher) for 10 min followed by washing three times with PBS. The stained cells were observed with a confocal microscope (Olympus Fv 1200 confocal laser scanning microscope).

### Glycosaminoglycan content analysis

Glycosaminoglycan formation on the surfaces was detected using alcian blue staining after 7, 14, and 21 days of culture, following the methods that have been described previously^25^. All surfaces were rinsed twice in PBS followed by fixation in cold (4 °C) acetone: methanol (1:1) solution for 3 min. Substrates were transferred to a 1% alcian blue solution in 3% acetic acid. The surfaces were incubated at room temperature in alcian blue for 30 min followed by three rinses in 3% acetic acid for 2 min each. After rinsing in deionized water for 2 min, the surfaces were allowed to dry for imaging or placed in a 1% sodium dodecyl sulfate solution for 30 min on a 200 rpm shaker plate to solubilize the alcian blue stain. The absorbance of the solubilized solution was measured at 605 nm.

### Real-time quantitative PCR analysis

Gene expression on the mRNA level was carried out to determine the occurrence of chondrogenesis. The genes examined in this study were type 2 collagen (COL2A1) and SOX9. Also, we used glyceraldehyde-3-phosphate dehydrogenase (GAPDH) as a housekeeping gene. RNA of hWJ-MSCs that have been grown on micropattern for 21 days was isolated using the SV Total RNA Isolation System protocol (Promega). Then the concentration and purity of RNA were measured using NanoDrop spectrophotometer. Complementary DNA (cDNA) synthesis was carried out using the GoTaq 2-Step RT-qPCR System protocol (Promega) followed by Real-Time PCR with the SYBR Green I assay method. The primers and the cycle condition used in this study can be seen in Table 1. The result of qPCR was analyzed following the 2^-ΔΔCT^ method described by Livak and Schmittgen (2001)^26^.

**Table 1:** Primer Sequence.

Genes	Primer Sequence	Annealing Temperature
GAPDH	5′ TCCTGTTCGACAGTCAGCCG 3′	61.5℃
	5′ CCCCATGGTGTCTGAGCGAT 3′	
COL2A1	5′ GAACCCAGAAACAACACAATCC 3′	
	5′ CATTCAGTGCAGAGTCCTAGAG 3′	
SOX9	5′ CAGTACCCGCACTTGCACAA 3′	
	5′ CTCGTTCAGAAGTCTCCAGAGCTT 3′	

### Statistical analysis

Statistical analysis was done using GraphPad Prism 9 software. For immunocytochemistry (ICC), the sample population was 2 for each group. For GAG and RT-qPCR assay, the sample population was 2 for each group with a technical repetition of 3 times. The normality test was performed using the D'Agostino & Pearson test method to determine the distribution of data. The quantitative data GAG and RT-qPCR assay were further analyzed, using a two-way analysis of variance (ANOVA) method for GAG assay, and a one-way ANOVA method for RT-qPCR assay with a significance of 95%. The analysis then continued with the Post-hoc Tukey HSD test to determine the significance subsets between every group at a 95% level (p-value < 0.05).

## Results and discussion

### Characterization of human Wharton’s jelly MSCs (hWJ-MSCs)

**Figure 2:**
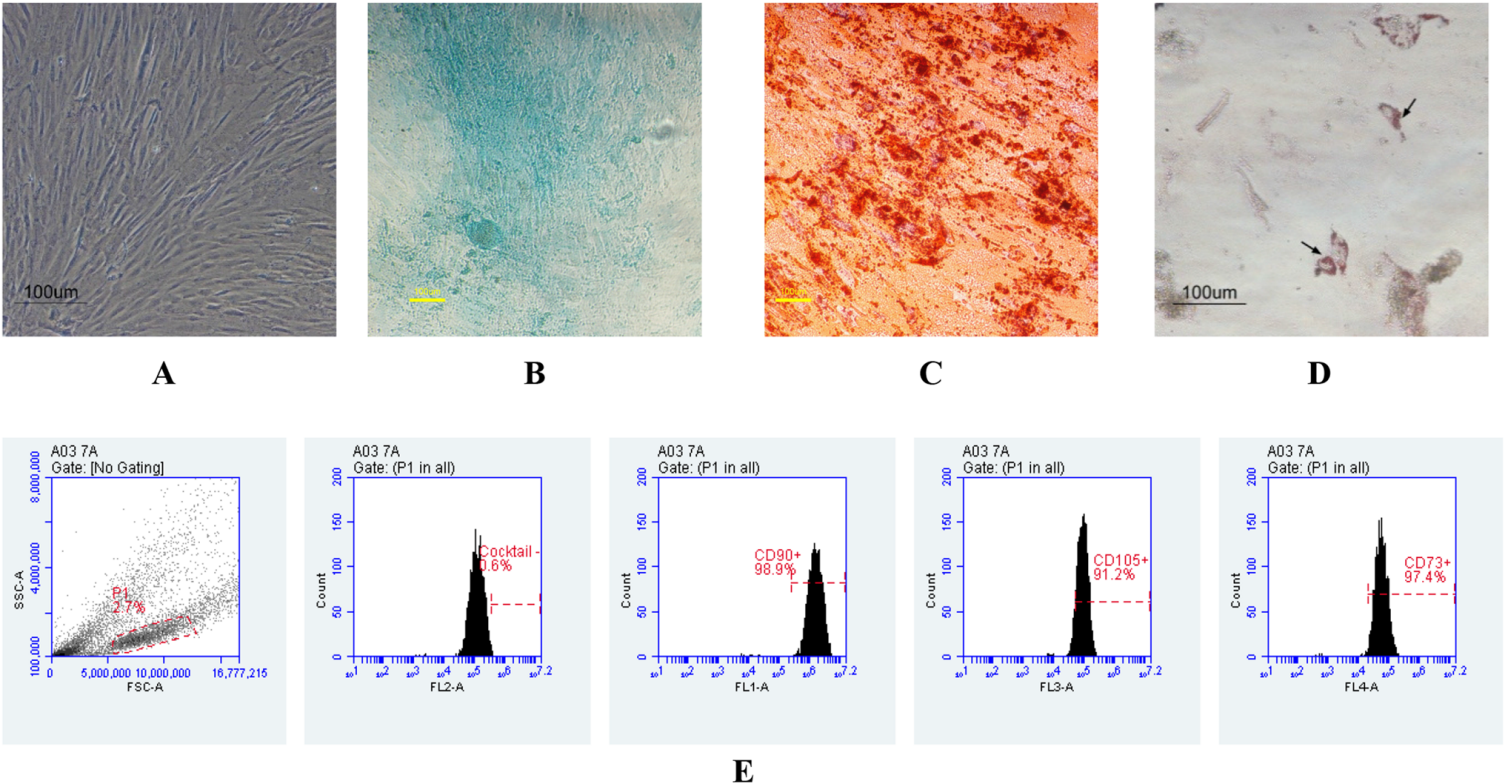
Characterization of Mesenchymal Stem Cell (MSC): plastic adherent (**A**), chondrogenic differentiation stained with Alcian Blue (**B**), osteogenic differentiation stained with Alizarin Red (**C**), adipogenic differentiation stained with Oil Red O (**D**), and analysis of positive MSC specific surface marker CD73 (97.4%), CD90 (98.9%), CD105 (91.2%), and negative marker (0.6%) (**E**). The black arrow showed the lipid droplet, scale bar = 100 µm.

hWJ-MSCs were characterized based on the Mesenchymal and Tissue Stem Cell Committee of the International Society for Cellular Therapy (ISCT). The features that define MSCs are plastic-adherent when maintained in standard culture conditions, express certain surface molecule markers, and can differentiate to osteoblasts, adipocytes, and chondroblasts in vitro^27^. The results showed that the cells used in this study were indeed MSCs because they can adhere to plastic, expressing surface molecule markers with > 95% CD105, CD 90, and CD 73 expression, and do not express negative markers (< 2%) such as CD 45, CD 34, CD 14, or IB CD, CD 74 or CD 19, HLA-DR. These results were contrast with MSC marker on fibroblast cells (non-MSC), showed that fibroblast cells only expressed 29.2% of CD 105 and 29.2% of CD90 (Supplementary Data 1, Figure A). hWJ cells can also differentiate into 3 lineages which are chondrogenic, osteogenic, and adipogenic fate that is characterized by the formation of glycosaminoglycan, calcium deposit, and lipid droplets, respectively (Fig. 2). Therefore, the hWJ cells used in this study met the MSC criteria.

### Micropattern fabrication using agarose microcontact printing (µCP)

**Figure 3:**
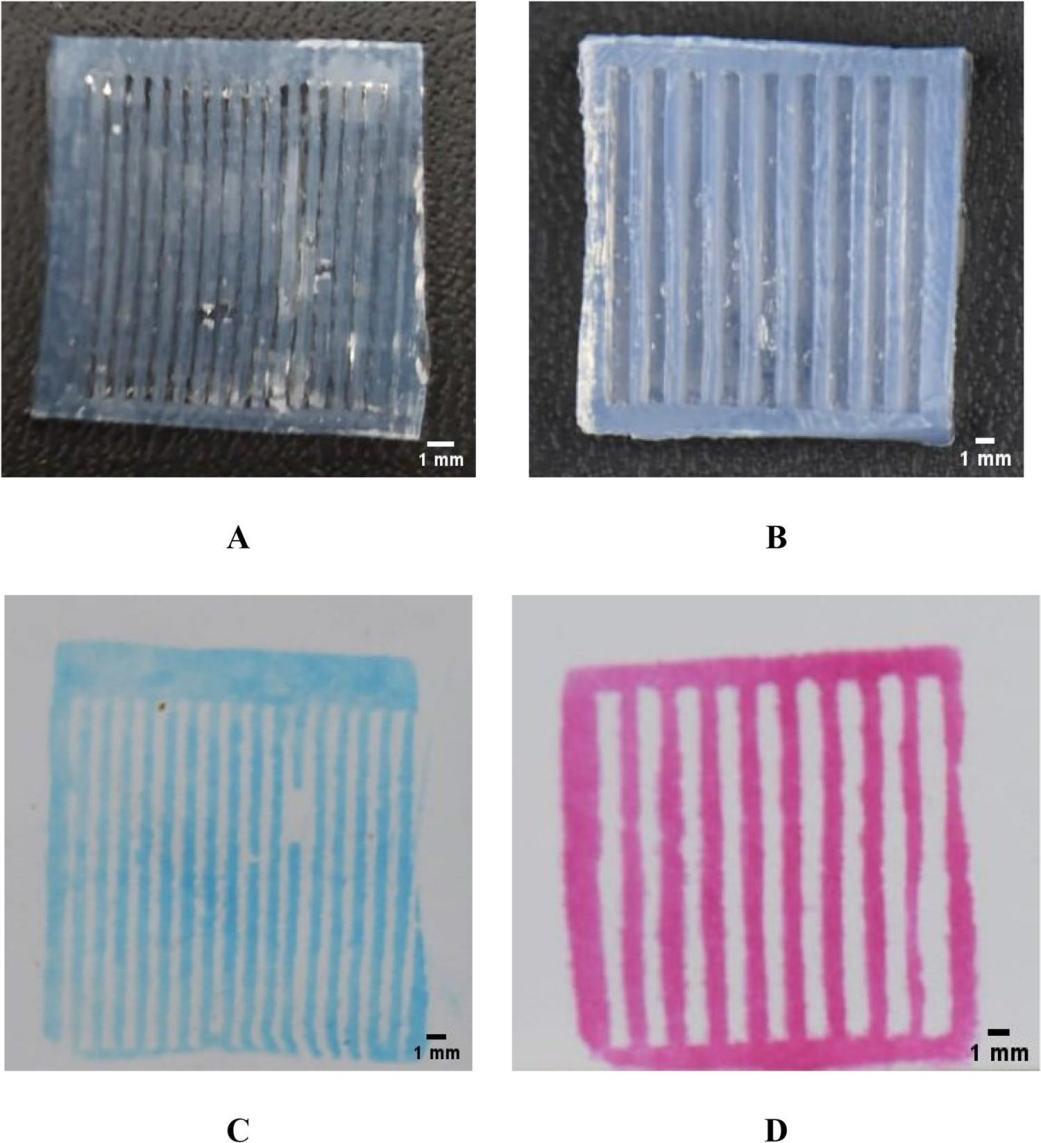
Agarose-micropattern stamp 500 µm (**A**), 1000 µm (**B**); and stamping results of 500 µm (**C**), 1000 µm (**D**) agarose-micropattern stamp using ink.

Agarose gel with a striped pattern of 500 µm and 1000 µm was successfully made from agarose 3% (w/v) (Fig. 3A,B). These agarose stamps have a dense consistency but can absorb ink well. The stamp can be stored in PBS at 4 °C and can be used repeatedly during the stamping procedure. The result also showed that the agarose stamp was effective in transferring ink to the substrate as evidenced by the ink pattern that was formed (Fig. 3C,D). Agarose gels have a lower hydrophobicity than other polymers such as PDMS^14^, making agarose gel easier to absorb ink and forming a homogenous pattern.

### Morphology of hWJ-MSCs cultured on micropattern surface

**Figure 4:**
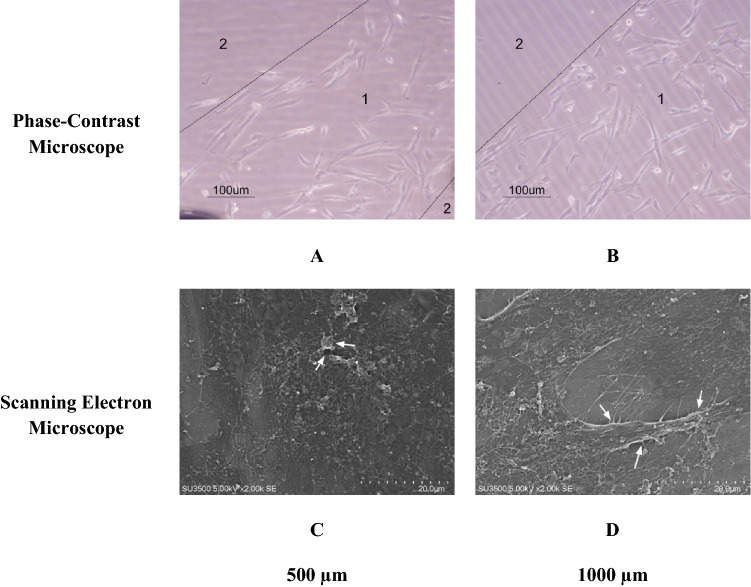
hWJ-MSCs morphology on micropattern surface 500 µm (**A**,**C**) and 1000 µm (**B**,**D**) fibronectin micropattern using µCP. The number represented fibronectin-coated area (1) and uncoated area (2), white arrow showed cytoplasmic protrusion.

Figure 4 showed that micropattern facilitates cell adhesion and spreading in a specific area. hWJ-MSCs were grown in the area coated with fibronectin pattern because it serves as an extracellular matrix and can interact with membrane proteins. The morphology of hWJ-MSCs was elongated and normally have cytoplasmic protrusions on the opposite side (Fig. 4A,B). Without micropattern substrate, the cells have a rounded shape that indicates poor adhesion with the surface. The membrane protein, such as integrin, is thought to bind RGD sequences of fibronectin which then leads to strong attachment. Scanning electron microscope (SEM) result showed that the cell shape has already flattened and has several protrusions that interact with the micropatterned substrate (Fig. 4C,D).

### *Argiope appensa* spidroin characterization

Physical properties of spidroin bioink were performed using zeta potential and viscosity. Also, the molecular weight of spidroin solution was estimated according to SDS-page electrophoresis.

**Figure 5:**
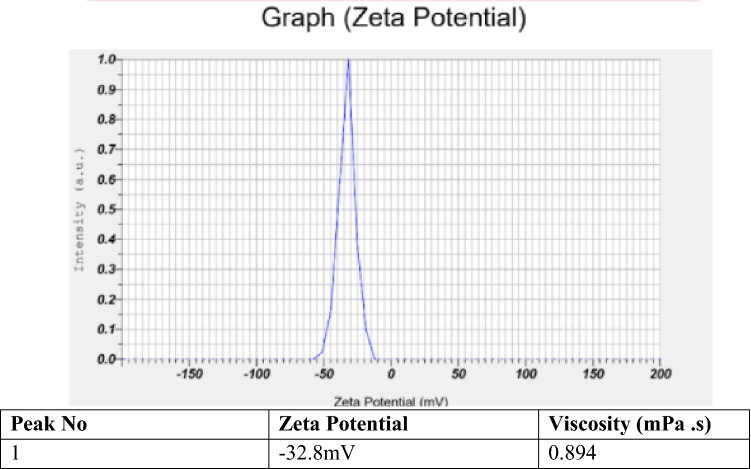
Spidroin’s zeta potential results, which has average value −32.8 mV therefore show a good particle stability.

**Figure 6:**
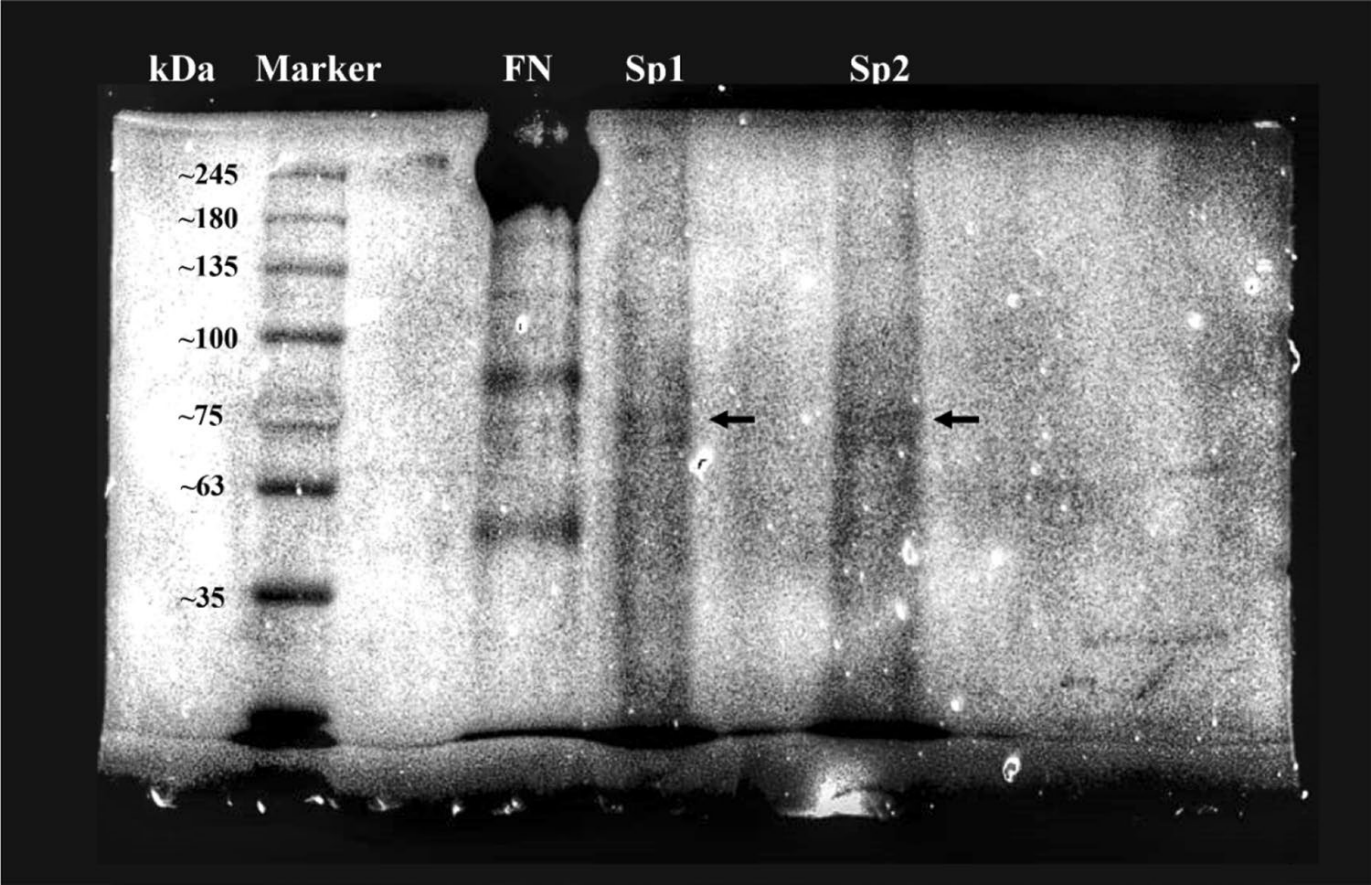
SDS-page analysis of Spidroin solution. FN: Fibronectin; Sp1: Spidroin sample 1; Sp2: Spidroin sample 2. FN used as a control. Black arrow is the protein target band.

Zeta potential is defined as the total charged that a particle has that will determine the ability of a particle to remain disperse or end up agglomerate. The value out of range −30 mV to + 30 mV is considered for particles to have an adequate repulsive force to retain their colloidal stabilities^28^. Spidroin’s zeta potential has average value −32.8 mV (Fig. 5) therefore show a good particle stability. The bioink shows other physical characteristics that it has viscosity lower than water (0.894 mPa.s). This low viscosity property is according to the SDS page result (Fig. 6) which shows a low kDA result. Low kDA on protein means the protein polymer is a short-chain polymer. This short-chain condition makes the interaction among two or more polymer chains are less strong and easy to be moved by any stirring process.

As shown in Fig. 6, the spidroin was present at around 75 kDa and fibronectin were ranging from 40 to 245 kDa. Compared to another species of *Argiope*, *Argiope aurantia,* the extract of spidroin derived from cylindrical gland silk fibers. From these gland, ten major proteins were resolved, with apparent molecular weights ranging from about 13 to over 200 kDa^29^. Spider silk that we used in this study was derived from spider web, not from the glands. Our result is quite unexpected, since it has different extraction method and source of the spider silk, but the molecular weight is still in the range of 13–200 kDa. These indicating spidroin solution might not degrade when extracted from spider silk.

### *Argiope appensa* spidroin as bioink for µCP micropattern

**Figure 7:**
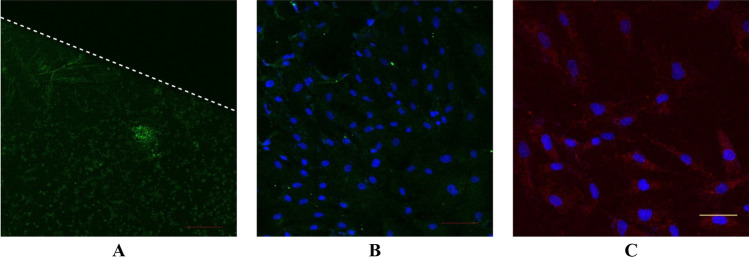
Immunocytochemistry (ICC) of RGD from spidroin micropattern without cell (**A**), with cell (**B**) and ICC of Integrin β1 from hWJ-MSC grown on spidroin micropattern (**C**). RGD appeared green, integrin β1 appeared red, and the nucleus appeared blue on images. Scale bar = 50 µm, white dashed line separated the spidroin micropattern (lower part) and non-patterned area (upper part).

This research studied the presence of the RGD sequences in spidroin bioink. Based on the ICC result (Fig. 7A), micropattern with spidroin bioink had RGD sequences, shown here as green fluorescence. When the micropattern was overgrown with cells, RGD appeared on the surface of the substrate even after 7 days of culture (Fig. 7B). This result confirmed the research conducted by Barlian et al. (2020) which showed that based on ICC, the 3D scaffold containing the spidroin mixture from *Argiope appensa* has RGD sequences^21^. In addition, several mechanical testing to characterize spidroin from *Argiope appensa* has been done, such as by using FTIR spectroscopy analysis^21^. Figure 7A,B proved that spidroin can be used as a bioink for micropattern production and the solubilization process did not damage the RGD sequences. Cell adhesion involves certain cell surface proteins called integrins, and these membrane proteins affect the organization of the cytoskeleton and responsible for mediating cell–matrix adhesion^30^. RGD sequences can bind to integrins through the β1 sub-unit^31^. Figure 7C showed that hWJ-MSC grown on spidroin micropattern expressed integrin β1. These results indicated that spidroin bioink was able to facilitate cell adhesion through interaction between RGD and integrin β1.

### Micropattern width effect on glycosaminoglycan matrix deposition

**Figure 8:**
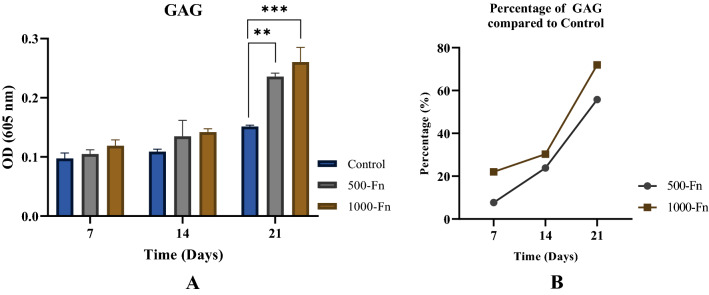
GAG accumulation of hWJ-MSCs grown on fibronectin micropattern (**A**) and percentage of GAG increased (**B**) on fibronectin (Fn) micropattern compared to control after 7, 14, and 21 days of culture (Control = non-coated coverslip, 500-Fn = 500 µm fibronectin micropattern, 1000-Fn = 1000 µm fibronectin micropattern). **denotes significant difference in GAG (p < 0.01), whereas ***(p < 0.001).

Glycosaminoglycan (GAG) levels of hWJ-MSCs on fibronectin micropattern were quantified on day-7, 14, and 21 (Fig. 8). GAG is one of the main functional elements of cartilage that plays an important role in homeostasis. Not only does it provide mechanical resistance to pressure, but GAG is also involved in signaling pathways that regulate cell adhesion, proliferation, and differentiation^32^. The results showed that the GAG levels in the micropattern treatment with the size of 500 µm and 1000 µm increased significantly compared to the control group on day-21. Several in vitro MSCs chondrogenesis studies showed that GAG levels will tend to increase from day-14 to day-28^33–35^.

A similar study on GAG content during MSCs differentiation with striped micropattern induction was done by Chou et al. (2013) using the same width size of 500 µm and 1000 µm, but the MSCs were cultured in chondrogenic medium condition^36^. Chou et al. found that micropattern treatment cells had higher GAG levels compared to the control group, however, GAG content on 500 µm and 1000 µm groups was the same^36^. This insignificant difference in GAG content can be caused by the usage of a chondrogenic medium that masked the effect of micropattern induction. Therefore, in this research, the cells were cultured in a basal medium without any chondrogenic supplementation. Our result showed that GAG levels did increase significantly on day-21 by as much as 56% for 500 µm and 72% for 1000 µm.

The role of spidroin micropattern from *Argiope appensa* to direct hWJ-MSC chondrogenesis has previously been investigated^22^. Hernando et al. showed that GAG level was highest in cells cultured on spidroin micropattern with 50 µg/ml spidroin concentration and 1000 µm micropattern width^22^. The spidroin concentration was the same as the fibronectin concentration used in this research (50 µg/ml). Our GAG result on fibronectin also proved that 1000 µm micropattern width could support chondrogenesis better than 500 µm size.

### Upregulation of SOX9 expression in both mRNA and protein by spidroin micropattern

**Figure 9:**
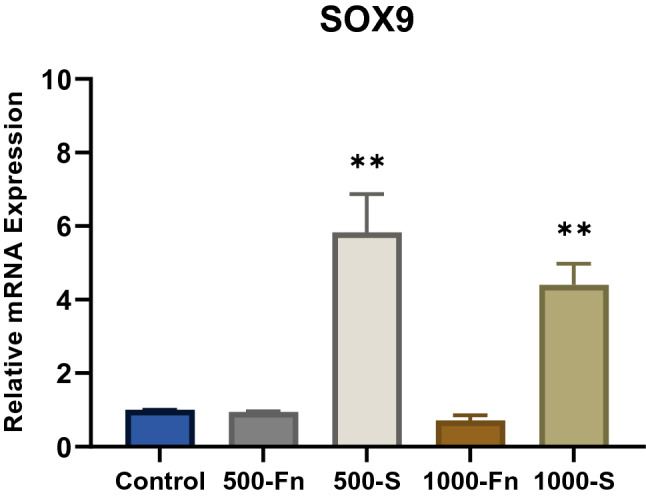
Relative SOX9 mRNA expression of hWJ-MSCs grown on fibronectin and spidroin micropattern after 21 days of culture (Control = non-coated coverslip, 500-Fn = 500 µm fibronectin micropattern, 500-S = 500 µm spidroin micropattern, 1000-Fn = 1000 µm fibronectin micropattern, 1000-S = 1000 µm spidroin micropattern). *denotes significant difference in relative mRNA expression (p < 0.05), whereas **(p < 0.01).

Spidroin micropattern treatment in both 500 µm and 1000 µm induced significantly high levels of SOX9 mRNA expression on day-21 (Fig. 9). On the other hand, fibronectin micropattern treatment has the same relative expression level as the control group. SOX9 expression is required for the initial step in mesenchymal condensation, differentiation, and proliferation of chondrocytes. Since SOX9 is a transcription factor that is essential and directly regulates collagen type II expression, SOX9 mRNA upregulation is thought to precede the expression of the COL2A1 gene^37^. However, during chondrogenesis, SOX9 activity is necessary for several successive steps^37,38^. The high relative expression of SOX9 mRNA in spidroin micropattern treatment during the late-stage of chondrogenesis (day-21) can be attributed to SOX9 function that inhibits proliferating chondrocytes transition into hypertrophy^38^.

**Figure 10:**
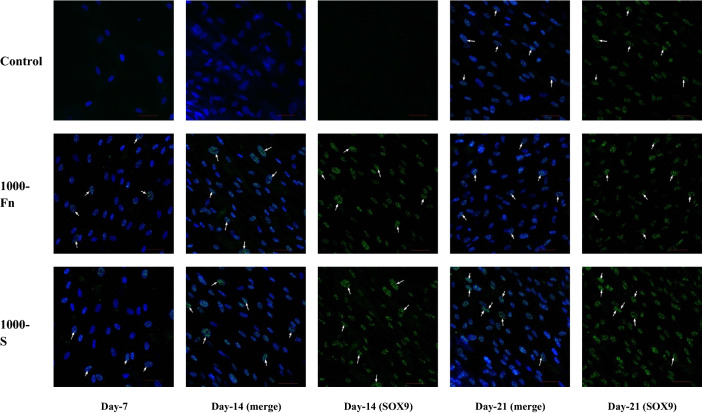
Immunocytochemistry (ICC) of SOX9 from hWJ-MSCs grown on spidroin and fibronectin micropattern after 7, 14 and 21 days of culture (Control = non-coated coverslip, 1000-Fn = 1000 µm fibronectin micropattern, 1000-S = 1000 µm spidroin micropattern). SOX9 appeared green and the nucleus appeared blue on images. Scale bar = 50 µm, white arrow showed SOX9 protein located inside the nucleus.

hWJ-MSCs grown on spidroin micropattern were able to maintain long-term induction of SOX9 gene expression compared to fibronectin. Studies showed that this long-term SOX9 activity could function to suppress RUNX2 expression, an osteogenic transcription factor, and therefore, prevents spidroin micropattern-induced cells from progressing into osteogenic fate^39,40^. SOX9 mRNA upregulation marks the onset of chondrogenesis. Afterward, the SOX9 mRNA level started increasing from day-14 and reached its peak on day-21^41^. Looking at the immunocytochemistry result on SOX9 protein, micropattern treatment accelerates the synthesis of SOX9 protein which can already be detected from day-7 (Fig. 10). Figure 10 also showed the presence of SOX9 protein on day-14 and 21.

Spidroin micropattern induces signaling pathways that regulate SOX9 expression, which in turn will advance the differentiation step towards chondrocytes. Our data showed that the increased level of SOX9 gene expression in spidroin treatment is correlated with an increase in SOX9 protein synthesis, as confirmed with immunocytochemistry. The SOX9 protein was detected in both spidroin and fibronectin micropattern from day-7 and localized inside the nucleus. Subsequently, SOX9 acts as a master regulator that plays an important role in the transcription of pro-chondrogenic genes such as type II collagen (COL2A1 gene) and core protein of proteoglycan^42^.

### Spidroin micropattern downregulates COL2A1 mRNA on late-stage chondrogenesis and induces protein expression

**Figure 11:**
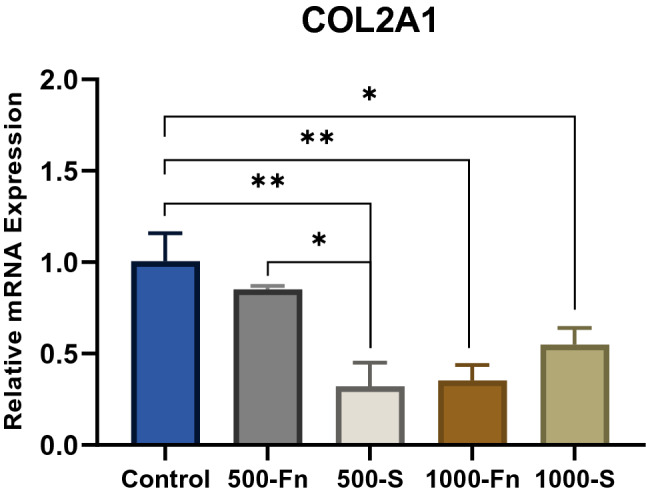
Relative COL2A1 mRNA expression of hWJ-MSCs grown on fibronectin and spidroin micropattern after 21 days of culture (Control = non-coated coverslip, 500-Fn = 500 µm fibronectin micropattern, 500-S = 500 µm spidroin micropattern, 1000-Fn = 1000 µm fibronectin micropattern, 1000-S = 1000 µm spidroin micropattern). *denotes significant difference in relative mRNA expression (p < 0.05), whereas **(p < 0.01).

COL2A1 gene expression data on day-21 showed that the relative expression of the spidroin and fibronectin micropattern treatment was significantly lower compared to the control group (Fig. 11). Throughout chondrogenic differentiation, the expression of collagen type II mRNA occurs during day-7 until day-11^43^. This explains why most of the COL2A1 mRNA in micropattern-induced cells on day-21 was decreased. The fact that the control group has higher COL2A1 relative mRNA expression on day-21, is an indicator of an incomplete or slow chondrogenic differentiation process^44^. This is further verified by analyzing the protein expression of collagen type II using immunocytochemistry. Both the spidroin and fibronectin treatment groups started synthesizing collagen type II protein on day-14, in comparison to the control group in which the collagen type II protein was not detected even on day-21.

**Figure 12:**
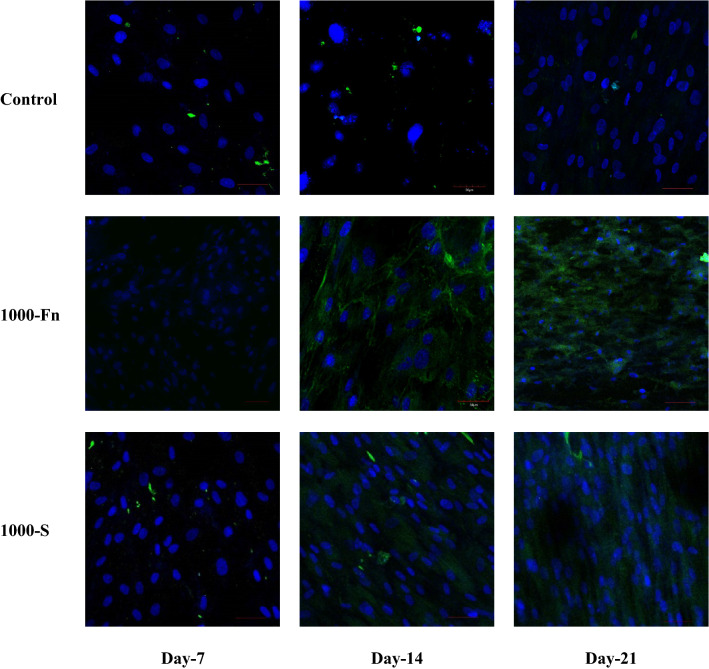
Immunocytochemistry (ICC) of Collagen type II from hWJ-MSCs grown on spidroin and fibronectin micropattern after 7, 14, and 21 days of culture (Control = non-coated coverslip, 1000-Fn = 1000 µm fibronectin micropattern, 1000-S = 1000 µm spidroin micropattern). Collagen type II appeared green and the nucleus appeared blue on images. Scale bar = 50 µm.

Based on the comparison of ICC results, it can be noted that SOX9 protein expression preceded collagen type II in micropattern treatment (Fig. 12). However, the relative expression of mRNA on day-21 did not show a correlation between SOX9 and COL2A1 gene expression. This may be due to SOX9 that regulates several steps of chondrogenic differentiation, and hence, a high level of SOX9 does not always increase COL2A1 mRNA nor collagen type II protein expression^45,46^. Additionally, long-term expression of collagen type II does not require SOX9^37^. Furthermore, the upregulation of the SOX9 gene in spidroin treatment occurred on late-stage chondrogenesis, in which collagen type II had already been synthesized.

**Figure 13:**
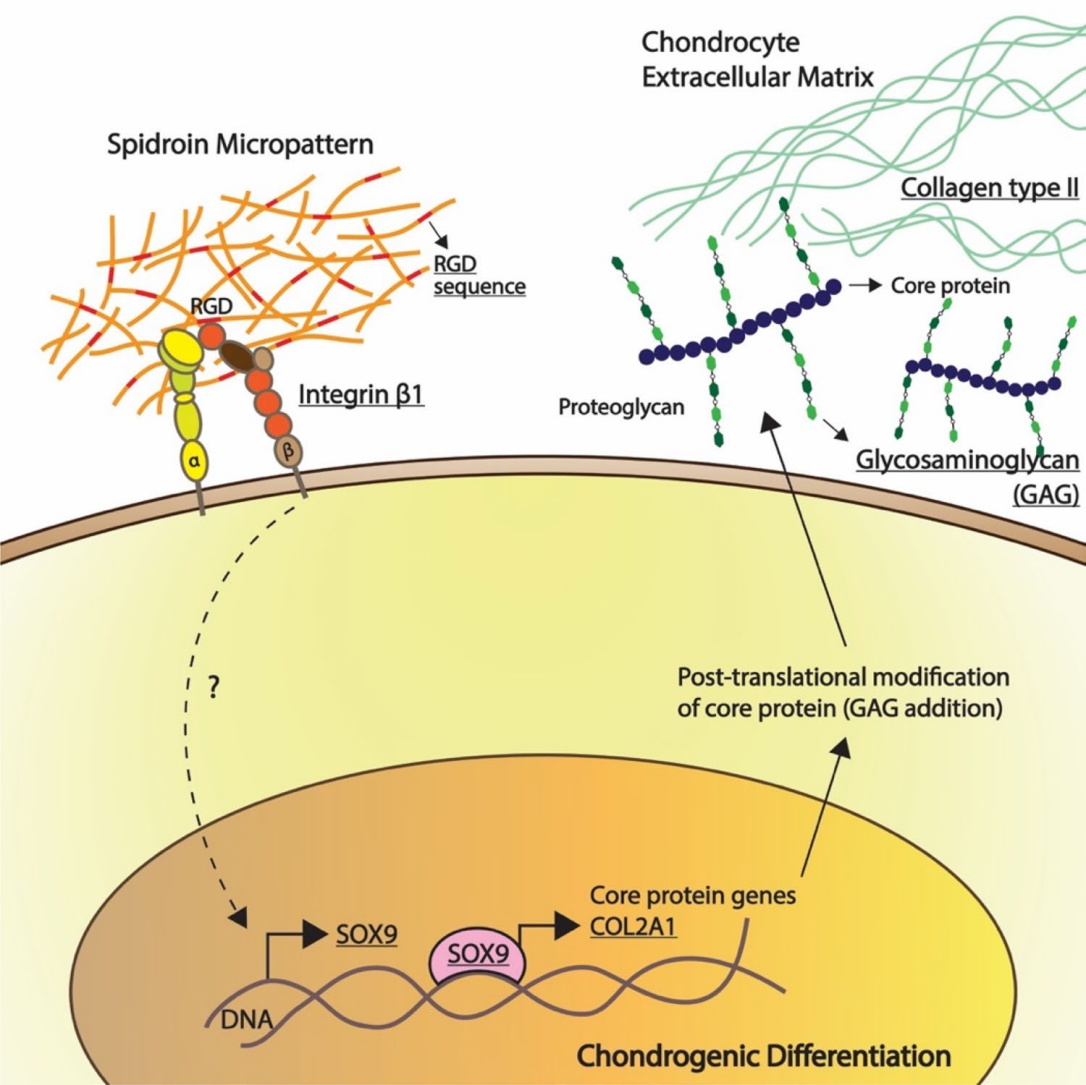
Mechanism of spidroin micropattern induction in chondrogenesis of hWJ-MSCs.

Spidroin micropattern was able to promote chondrogenesis of hWJ-MSCs in vitro without chondrogenic inducers such as the TGF-β superfamily. Biomaterials containing RGD sequences can induce differentiation because the interaction between MSCs and RGD sequences mimics the cell microenvironment during chondrogenesis of limb bud development^47^. Figure 13 showed the mechanism of spidroin micropattern on chondrogenesis. RGD sequences on spidroin interact with integrin β1 located on the cell membrane. Which then activates the expression of the SOX9 gene, the master regulator of pro-chondrogenic genes. Subsequently, SOX9 protein acts as a transcription factor and increases the expression of the COL2A1 gene that encodes collagen type II protein. Spidroin also increases GAG content which is a part of proteoglycan molecules that consists of core protein attached to the GAG chain.

Even though the exact signaling cascade from spidroin micropattern interaction with integrin β1 to the elevated expression of the SOX9 gene remains unclear, previous studies showed that integrin-linked kinase (ILK) and p38 MAPK might be involved. Perera et al. (2010) noted that integrin activation leads to phosphorylation of integrin-linked kinase (ILK), which in turn phosphorylate p38 MAPK^48^. The MAPK signaling network is thought to control the response of micropattern induction on chondrogenic differentiation. Studies showed that p38 MAPK protein is responsible for regulating SOX9 expression level by maintaining the half-life stability of SOX9 mRNA^49,50^.

Another factor that contributes to micropattern induction that resulted in chondrogenesis is the micropattern 1000 µm width. The micropattern size provides optimal cell-to-cell contact and promotes aggregation leading to chondrogenesis. Several studies proved that the membrane protein that facilitates interaction between cells and has a vital role in chondrogenic mesenchymal condensation is N-cadherin^51,52^. According to previous studies, the formation of the N-cadherin complex with adjacent cells resulted in the phosphorylation of free β-catenin. Once phosphorylated, it becomes inactivated and will bind to the membrane adhesion complex. This β-catenin inactivation prevents β-catenin from inducing SOX9 degradation via proteasome that will inhibit chondrogenesis^53–55^. Thus, based on other studies, micropattern width is thought to influence chondrogenic differentiation by a different signaling cascade from spidroin.

Overall, this research showed that spidroin can be used as a bioink for micropattern and induced chondrogenesis at the same level as fibronectin. Fibronectin has long been known as the ideal substrate for micropatterning due to the availability of RGD sequences in the protein. This study proved that *Argiope appensa* spidroin contains RGD sequences and enhances the hWJ-MSCs differentiation process as shown in the ICC and RT-qPCR results. Micropattern treatment restricts cells to a particular area, which leads to aggregation, a crucial step in chondrogenesis. Spidroin micropattern thus can be seen as a new approach for cartilage tissue engineering, to accelerate the chondrogenic differentiation of MSCs. Future study is required to elaborate more regarding the underlying molecular pathway that mediates the signals from integrin β1 to the expression of SOX9.

Thus far, micropatterning of cells is still limited to 2D surfaces. In this study, the micropatterning was used to differentiate hWJ-MSCs within a shorter period and determine the size of the pattern. Micropattern itself prevents further dedifferentiation of chondrocytes when cultured in 2D substrate^56^. However, the structure of 3D native cartilage is different from micropatterned chondrocyte culture. Recent research showed that we can create micropattern surface in cell sheet technology and eventually stacking it to form complex 3D shapes^57^. Multilayered cell sheets can be formed by magnetic technology such as through the attraction of MNP-labelled cells. However, this needs further research regarding the maintenance of this 3D structure, for example oxygen, nutrient, and also metabolite exchange.

## Supplementary Information

Supplementary Information 1.

Supplementary Information 2.
