# Supplementary material for: Spidroin striped micropattern promotes chondrogenic differentiation of human Wharton’s jelly mesenchymal stem cells
Source: Sci Rep. 2022 Mar 22;12:4837. doi: 10.1038/s41598-022-08982-8 (PMC8941093; doi:10.1038/s41598-022-08982-8)
Supplement: Supplementary file 1 — Supplementary Information 1. [file 41598_2022_8982_MOESM1_ESM.pdf]

Supplementary Data 1.

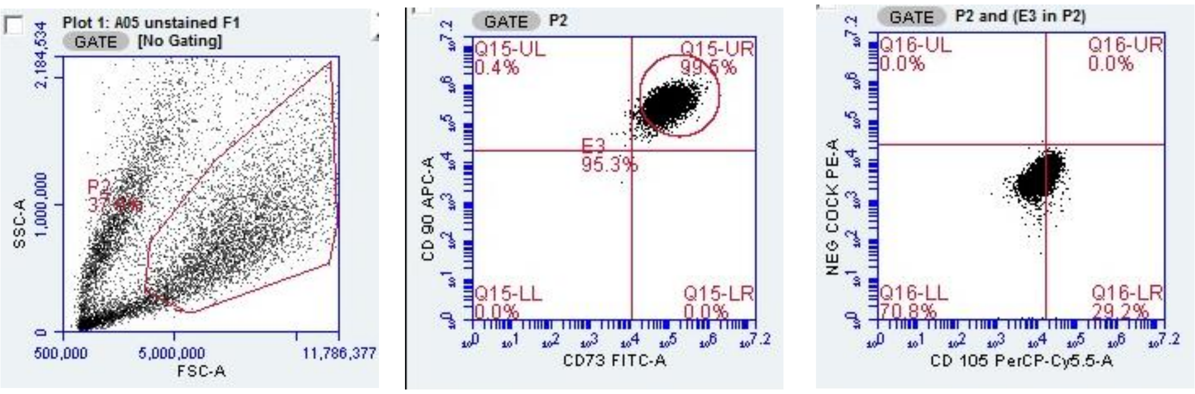

Figure A. MSC specific surface marker for fibroblast cells (non-MSC) showed expression of CD 73 (99.5%), CD 90 (99.9%), CD 105 (29.2%), and negative marker (0%).
